# Signalling disease outbreaks: cost-effectiveness analysis of early warnings and response systems in the case of dengue control

**DOI:** 10.1186/2047-2994-4-S1-P235

**Published:** 2015-06-16

**Authors:** H-C Stahl, V-M Butenschön, D Sonntag, A Farlow, International Research Consortium on Dengue Risk Assessment, Management and Surveillance (IDAMS)

**Affiliations:** 1 Médecine Orpheline, France; 2 Institute of Public Health, Mannheim, Germany; 3 University of Oxford, UK

## Introduction

Early warnings and response systems (EWS) are of up-most importance to identify disease outbreaks and to initiate life saving interventions. Up to date disease and vector-surveillance function as early warning systems to detect dengue outbreaks at early stages, so as to respond early to outbreaks and save resources. Currently, there is no vaccine or causal treatment for dengue fever, and methods to reduce the life saving burden relying on EWS, and early warning responses. This paper investigates the cost-effectiveness of EWS for Brazil, based on a decision analytical model.

## Objectives

Assess the cost-effectiveness of early warnings and surveillance systems of infectious diseases: the case of dengue.

## Methods

Based on the SINAN database (national notifiable diseases information system), we extracted the severity distribution of dengue illness. WHO Unit Costs were adjusted to I$, to create a severity-based cost-function for direct costs of dengue illness. Effects were presented as DALYs averted. Costs and Severity were adjusted to the Oxford risk map incidence number. A decision-tree model including 3 response efficacies (70% outbreak prevention, 50%, and 30%) was constructed to assess the cost-effectiveness of an early warning system at a state-level for Brazil. Tornado diagrams were performed to investigate the impact of chosen variables on the expected ICER value.

## Results

With a sensitivity of 0.57, false alarm rate of 0.12, and a 70% chance of preventing an outbreak, the implementation of an EWS is very cost-effective (ICER < 1 GDP/capita) in 25/27 states in Brazil. For the medium efficacy EWS, 22/27 states would benefit from its implementation, and for the lower efficacy early warning responses, only 8 states profit from an EWS. The expected ICER value was among others influenced by the costs of the EWS, the response costs, and the false alarm rate.

## Conclusion

Implementing early warning systems with a medium or a high efficacy showed efficiency gains, which is cost-effectiveness. We hereby provide a model, which can be adjusted by imputing country specific data, but to fully assess their impact, more studies need to be conducted on the adequacy and feasibility, on reliable alarm signals and especially the outcome of interventions.

## Disclosure of interest

None declared.

